# Hypothalamic hamartoma with pubertas precox and gelastic seizure in a boy (Case Report)

**DOI:** 10.1186/1687-9856-2013-S1-P196

**Published:** 2013-10-03

**Authors:** Iman Hendarman, Aditiawati, Msy Rita Dewi Arifin

**Affiliations:** 1 Department of Child Health, Faculty of Medicine Sriwijaya University/Dr. Mohammad Hoesin Hospital, Palembang, Indonesia

## 

Hypothalamic hamartoma is a rare neoplastic heteropia caused by organic developmental failure. The most common clinical findings in hypothalamic hamartoma are pubertas precox with or without gelastic seizure, and behavioural disturbance. The aim of this case report to inform a rare case of hypothalamic hamartoma with pubertas precox and gelastic seizure in a boy.

A 5 year and 7 month old boy, admitted to the hospital with a chief complain of premature pubic hair growth and frequent sudden laughing without apparent reason which seemed to be forced, uncontrolled, irregular without deteroratoin of consciousness. Physical examination found accelarate height growth with appropriate proportion. Tanner pubertal status was A_1_P_2_G_2-3_, and neurological status was normal. Laboratory finding: FSH: 0,5 mlU/ml, LH: 0,85 mlU/ml, testosterone: 239,20 ng/dL, 17α-hydroxyprogesterone: 107 ng/dl. Cranial CT scan and MRI showed a space occupying lession in suprasellar region which was suspected as a tubercinerium hamartroma. Bone age result was appropriate for an 8 year and 6 month old boy. He was treated with leuprorelin 1x3,75mg intramuscular every 4 week and valproic acid 2x200mg. After 3 years evaluation, patient’s Tanner pubertal status was A_1_P_2_G_2-3_ and the bone age was appropriate for an 13 years old boy. No gelastic seizure.

Leuproleline effectivity as a therapy for pubertas precox with hypothalamic hamartoma is still a controversion, so management through surgical approach may be considered, and valproic acid has a good effectivity for gelastic seizure in this case.

